# Preparation, Optimization, and Screening of the Effect of Processing Variables on Agar Nanospheres Loaded with Bupropion HCl by a D-Optimal Design

**DOI:** 10.1155/2015/571816

**Published:** 2015-05-19

**Authors:** Jaleh Varshosaz, Mohammad Reza Zaki, Mohsen Minaiyan, Jaafar Banoozadeh

**Affiliations:** ^1^Department of Pharmaceutics, Novel Drug Delivery Systems Research Centre, School of Pharmacy and Pharmaceutical Science, Isfahan University of Medical Sciences, Isfahan 81746-73461, Iran; ^2^Exir Pharmaceutical Company, Borujerd, Iran; ^3^Department of Pharmacology, School of Pharmacy and Pharmaceutical Science, Isfahan University of Medical Sciences, Isfahan 81746-73461, Iran

## Abstract

Bupropion is an atypical antidepressant drug. Fluctuating in its serum levels following oral administration of immediate release dosage forms leads to occasional seizure. The aim of the present work was designing of sustained release bupropion HCl nanospheres suited for pulmonary delivery. Agar nanospheres were prepared by transferring the w/o emulsion to solid in oil (s/o) suspension. Calcium chloride was used as cross-linking agent and hydroxypropyl *β*-cyclodextrin (HP*β*CD) was used as permeability enhancer. A response surface D-optimal design was used for optimization of nanospheres. Independent factors included in the design were calcium chloride percent, speed of homogenization, agar percent, and HP*β*CD percent. Optimum condition was predicted to be achieved when the calcium chloride was set at 7.19%, homogenization speed at 8500 rpm, agar content at 2%, and HP*β*CD at 0.12%. The optimized nanoparticles showed particle size of 587 nm, zeta potential of −30.9 mV, drug loading efficiency of 38.6%, and release efficiency of 51% until 5 h. The nanospheres showed high degree of bioadhesiveness. D-optimal response surface method is a satisfactory design to optimize the fabrication of bupropion HCl loaded agar nanospheres and these nanospheres can be successively exploited to deliver bupropion in a controlled manner for a sufficiently extended period.

## 1. Introduction

Bupropion is an atypical short-acting aminoketone antidepressants drug [1, 2] which inhibits the reuptake of dopamine and norepinephrine [3]. It was patented for the first time in 1974 [4] and released onto the world market in 1985 but was briefly withdrawn due to seizures incidence. It was reintroduced in 1989 after the daily recommended dose was reduced to lower seizure likelihood [3].

To address the dose-related risk of seizures associated with high peak concentration of the drug following oral administration, bupropion hydrochloride is administered in divided doses or as sustained release dosage forms [1, 2, 5, 6].

Bupropion has numerous therapeutic indications including depression [7], smoking cessation [8], sexual dysfunction [9], obesity [10], attention deficit hyperactivity disorders [11], and seasonal affective disorders [12]. Following oral administration of immediate release forms of bupropion, peak plasma concentration is usually achieved within 2 hours. The half-life of the postdistributional phase of bupropion ranges from 8 to 24 hours. Bupropion has also a relatively high volume of distribution of 18.6 L/kg [2, 13].

Following absorption from gastrointestinal tract, bupropion undergoes extensive first-pass metabolism with oral bioavailability of only 5% resulting in formation of metabolites which are less potent than the parent molecule while being more convulsion inducing [14, 15].

To overcome the shortcomings of currently available dosage forms, that is, (i) fluctuation in plasma level due to immediate release forms and (ii) conversion of parent molecule to its metabolites through first-pass effect, it is worth it to try other routes of administration in context of sustained release of the drug more common form of which being nanoparticles.

Among various natural polymers available, agar and agarose (fractions of agar) have been used to prepare micro- and nanoparticles by several workers [17–21]. Agarose hydrogel as nanoparticulate drug delivery system has the advantage of being administrable via different parenteral ways such as subcutaneous, intramuscular, intravenous, and pulmonary routes [17].

**Figure 1:**
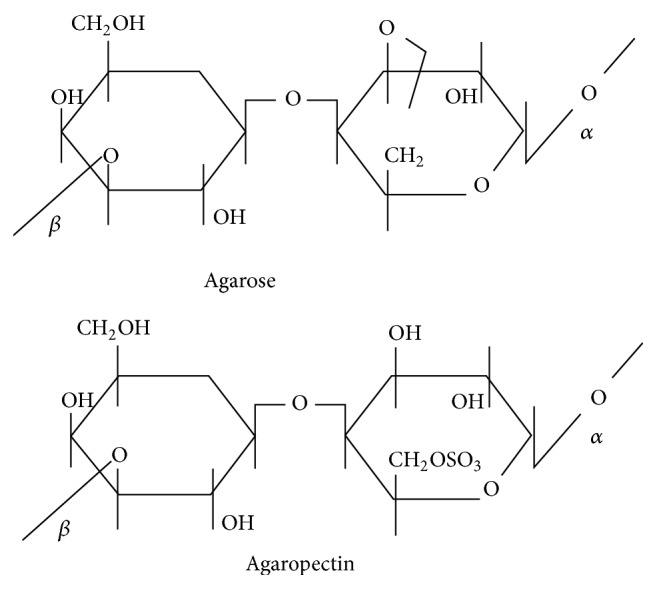
Chemical structure of subunits of agar including agarose and agaropectin.

Agar is composed of two polysaccharides, agarose, and agaropectin (Figure 1). Agarose contains 1, 3 linked* D*-galactose and 1, 4 linked 3, 6 anhydro* L*-galactose units, with very few hydroxyls being sulphated, useful as a material for the gel formation. Agaropectin is a more complex structure than agarose, containing in addition to* D*-galactose and 3, 6 anhydro galactose units,* D*-gluconic acid, pyruvic acid, and a much higher proportion of sulphate ester groups [20].

Agar shows some interesting physical properties. It is not soluble in cold water but is soluble in boiling water. Its solution forms thermally reversible hydrogels while being cooled down below its gelation temperature (31–36°C). The reversible gel-to-sol transition for the agarose hydrogel does not occur below the melting point (65–85°C) [22].

In production of nanoparticles, different techniques have been used based on creating w/o emulsion, the aqueous phase of which consists of polymer solution dispersed in an oily continuous phase which is corn oil [17], soybean oil [18], liquid paraffin [20, 23], or methylene chloride [19]. Some researchers have tried to cover the agar microparticles simultaneously with a layer of PLGA making use of a phase separation technique [19].

Some reported methods for manufacturing of agar particles mentioned in literature are emulsification [24, 25], spraying-gelation [26], membrane emulsification [13], and nanoprecipitation [21].

Mucoadhesion is the other feature of agar which makes it an appropriate candidate for use as the matrix of nanoparticles [20].

Fabrication of nanoparticles loaded with various therapeutically active molecules is currently under extensive research by scientists worldwide. Nanocarriers used in pulmonary delivery of systemic and local drugs are mainly made of natural or synthetic polymers. Agar, a natural polymer, has not received the appropriate attention it deserves by the researchers. A limited number of works are published in the literature making use of agar or agarose to deliver drugs in the form of nano- or microparticles, none of them related to the respiratory tract. Wang and Wu [19] entrapped protein molecules in agarose which was further encapsulated within poly(lactic-co-glycolic acid) (PLGA) microspheres. These potentially could be administered in form of different dosage forms via various delivery routes. In another study, the same authors prepared agar nanoparticles, employing ovalbumin as a model drug [17]. These nanoparticles were declared as a possible vehicle for sustained delivery of peptides and proteins. Ju et al. [18] prepared agar nanoparticles containing protein molecules to be delivered from a suitable route. Manjunatha et al. [23] prepared sustained release diclofenac containing agar beads for application in delivery of the drug. Linghui et al. [27] proposed precipitation method for fabricating nano-agar particles. Sustained release of mucoadhesive agar microspheres loaded with metformin HCl for oral delivery of metformin was tried by Bera et al. [20].

On the other hand, pulmonary delivery of various therapeutic molecules in context of polymers other than agar is being attempted currently by several workers, but pulmonary delivery of drugs using agar nanocarrier has not been reported so far to the best of our knowledge. So considering the unique features of agar and shortcomings of oral delivery of bupropion HCl mentioned through the previous paragraphs, it is worth trying out to administer bupropion HCl via pulmonary route in the form of drug loaded agar nanospheres.

The main drawback of all reported methods for preparation of agar nanoparticles is their severe stickiness which prevents their wide use. In the present study, we have tried to design nanoparticles of bupropion for pulmonary administration by incorporating it into agar nanoparticles adopting the above mentioned general method with some modifications to prevent their stickiness and obtaining freely reconstituting sediments. Calcium chloride which bears a relative negative charge was used for cross-linking of the agar nanoparticles to retard the release rate of bupropion hydrochloride. As these nanoparticles are intended to be administered via pulmonary route, hydroxypropyl beta cyclodextrin (HP*β*CD) which shows the lowest cell toxicity on pulmonary epithelial cell lines and is the only modified *β*CD cited in the FDA list of Inactive Pharmaceutical Ingredients [28] was also used as a penetration enhancer.

## 2. Materials and Methods

### 2.1. Materials

Agar was obtained from Narico (Germany), liquid paraffin was obtained from Golnoosh Company (Iran), hydroxypropyl beta cyclodextrin which is not a hazardous or not classified as dangerous substance was purchased from Sigma (US), Bupropion HCl (99.8% pure) was purchased from Dipharma (Italy), and calcium chloride and methanol HPLC grade were purchased from Merck Chemical Company (Germany). All other reagents were of analytical grades.

### 2.2. Preparation of Nanospheres

Agar (100, 150, and 200 mg) was added to 10 mL of water. The resultant suspension was heated up to boiling temperature to dissolve the agar and then cooled down to 45°C keeping the vessel covered meanwhile to prevent the loss of water. Calcium chloride, HP*β*CD, and bupropion HCl previously dissolved in appropriate amounts of water (according to Table 1) were added at this temperature and mixed for three minutes. The resultant solution was added under homogenization to 40 mL of liquid paraffin previously warmed up to 40°C. After homogenizing at predetermined speeds by homogenizer (Ika T25 basic, Janken and Kunkel GmbH, Germany) for 2 minutes, the suspension was cooled down below 20°C by immersing the vessel in ice water bath. Centrifugation at 8000 rpm for 5 minutes was performed to settle the nanospheres. The sediment was washed three times by redispersing in 5 mL of methylene chloride. The final sediment was dispersed in 3 mL of ethanol, added to 3 g of mannitol, mixed thoroughly to get a homogenized paste, and left overnight to get air dried.

### 2.3. Morphological Study of Nanospheres

The scanning electron microscopy (SEM) studies were conducted on a Philips XL 30 instrument (USA) operating at 26 keV.

### 2.4. Zeta Potential, Particle Size, and Size Distribution Measurement

Particle size and zeta potential measurements were performed by Malvern ZetaSizer (Model 3000 NS, UK).

### 2.5. Drug Loading Efficiency Determination

Accurately weighted 40 mg of nanoparticles was dispersed in 20 mL of phosphate buffer (pH 7.2), shaken (100 rpm) for 24 hours, and assayed after filtration through 0.22 *μ*m syringe filter by HPLC method according to Loboz et al. [29] method. The analysis was performed by a Knauer HPLC system with Chromgate Software version 3.1 equipped with a binary pump, Smartline-1000-1 and Smartline-1000-2, and a UV detector (Smartline-UV-2500, variable wavelength, programmable, Berlin, Germany), an online solvent vacuum degasser, and a manual sample injector. Analysis was carried out on a C18 column (25 cm × 4.6 mm, particle size of 5 *μ*m) from Agilent (USA). The mobile phase consisted of methanol and 0.05 M phosphate buffer adjusted to pH 5.5 with phosphoric acid (85%) before addition of methanol (45 : 55 v/v). The flow rate was maintained at 1.0 mL/min at ambient temperature.

Loading efficiency (LE) was calculated according to the following equation:

(1) LE%=Analyzed weight of drug in nanoparticlesTheoretical weight of drug loaded in the system ×100.

### 2.6. *In Vitro* Release Study

Nanospheres equivalent to 2.5 mg of bupropion HCl were dispersed in 3 mL of phosphate buffer (pH 7.4), decanted in dialysis bag, and placed in 200 mL of 37°C buffer. At predetermined time intervals, 3 mL of release medium was withdrawn and analyzed spectrophotometrically at 298 nm to determine the released amount of bupropion HCl. The withdrawn samples were replaced by 3 mL of fresh 37°C buffer.

Release efficiency (RE) at 300 minutes was calculated according to the following equation for each formulation:

(2) $$\begin{array}{c} \text{R}{\text{E}}_{300}\%=\frac{\int_{0}^{300}ydt}{y_{100}\times t}\times 100, \end{array}$$

where *y* is the released percent at time *t*.

### 2.7. Optimization Method

D-optimal design minimizes the determinant of the (*X*′*X*)^−1^ matrix. They are built algorithmically to provide the most accurate estimates of the model coefficients. In this study, Design-Expert Software (version 7.0.0, Stat-Easc, Inc., Minneapolis, MN, USA) was used to develop a D-optimal response surface methodology (RSM) design based on the independent factors of calcium chloride percent (*X*_1_ or C), homogenization speed (*X*_2_  or  S), agar percent (*X*_3_ or A), and HP*β*CD percent (*X*_4_ or H). Based on the D-optimal design, a total of 25 experiments were performed. This allowed the choice of the best model from the linear model 3a, a two-factor interaction model 3b, and a quadratic model 3c based on the* F*-value derived from ANOVA, and the *R* ^2^, predicted *R* ^2^, and adjusted *R* ^2^. Consider

(3a) $$\begin{array}{c} Y_{i}=\beta_{0}+\beta_{1}X_{1}+\beta_{2}X_{2}+\beta_{3}X_{3}+\beta_{4}X_{4}, \end{array}$$

(3b) Yi=β0+β1X1+β2X2+β3X3+β4X4 +β12X1X2+β13X1X3+β14X1X4 +β23X2X3+β24X2X4+β34X3X4,

(3c) Yi=β0+β1X1+β2X2+β3X3+β11X12 +β22X22+β33X32+β44X42+β12X1X2+β13X1X3 +β14X1X4+β23X2X3+β24X2X4+β34X3X4.

In these equations, *Y*_*i*_ is the predicted value on any of the chosen measured responses (i.e., *Y*_1_: particle size, *Y*_2_: zeta potential, *Y*_3_: drug payload efficiency, and *Y*_4_: percentage of release efficiency after 5 hours), *β*_0_ is an intercept, *β*_1_, *β*_2_, *β*_3_, and *β*_4_ are linear coefficients, *β*_12_, *β*_13_, *β*_14_, *β*_23_, *β*_24_, and *β*_34_ are the coefficients of the two-way interaction terms, and *X*_1_, *X*_2_, *X*_3_, and *X*_4_ are the independent experimental variables which were selected based on the results from a preliminary study. Design-Expert version 7 (Stat-Ease, USA) was used for statistical analysis of the data. The *P* values less than 0.05 were considered to be statistically significant. In cases of getting insignificance models (*P* > 0.05), model reduction was performed to get a significant one.

For each response, the model suggested by the software was used to fit the data and the mathematical equation suggested by the software was solved to get the optimal points. A two-factor model was used to assess the relationship between the studied variables with the particle size and zeta potential while, a quadratic model for release efficiency and a linear model for loading efficiency. In cases of getting insignificance models (*P* > 0.05), model reduction was performed to get a significant one.

Optimization of response factors was performed for minimizing the particle size while maximizing the absolute value of zeta potential, loading efficiency, and RE_300_. Solution provided by the software with the greatest desirability was chosen as the optimum condition and, after executing the experiment based on the suggested values for the independent factors, the real responses were compared with the predicted ones and error percentages were calculated to evaluate the predictive ability of the models.

Design expert software produced 25 runs; the composition of each run is given in Table 1.

### 2.8. Mucoadhesion Evaluation

The extent of mucoadhesion was measured according to Varshosaz and Dehghan [30] method with some modification. Briefly, the calibration curve was produced by measurement of the absorbance of different concentrations of mucin in acetate buffer (pH 4.5) at *λ*_max⁡_ = 500 nm. Nanoparticles were added to acetate buffer (pH 4.5) containing mucin (1 mg/mL) and were mixed for 1 hour. After centrifugation, the absorbance of the supernatant was determined at the same *λ*_max⁡_. The concentration of free mucin was calculated from the calibration curve. The percent of mucin adsorbed on the surface of nanospheres which is an indicator of the extent of mucoadhesion was calculated according to the following equation:

(4) $$\begin{array}{c} A=\left(1-C_{\text{free}}\right)\times 100, \end{array}$$

where *A* is the percent of adsorbed mucin and *C*_free_ is the concentration of free mucin in the supernatant in mg/mL.

The optimized formulation was prepared with various concentrations of hydroxypropyl *β*-cyclodextrin (0, 0.5 and 1%) and the mucoadhesion of the optimized nanospheres was measured in acetate buffer. Also the mucoadhesion of the optimized formulation was checked in the simulated lung fluid (Gamble's solution) [16] which its composition is mentioned in the following paragraph.

### 2.9. Stability Test of Nanoparticles

Considering that bupropion release percentage from the optimized nanospheres after 5 hours was about 50%, the release data were fitted with different kinetic models including the first order release kinetic [*M* = *Q*_0_(1 − *e* ^−*Kt*^)], the zero order (*Q* = *Q*_0_ + *Kt*), and Higuchi model (*Q* = *Kt* ^1/2^). In these equations, *M* is the amount of drug remaining to be released in time *t*, *Q* is the amount of drug released at time *t*, *Q*_0_ is the amount of initially loaded drug, and *K* is the drug release constant. The best model which was better fitted to the release data was chosen according to the highest regression coefficient and the time required for the total release of the drug was anticipated according to that model. The *r* ^2^ values for these models were 0.9411, 0.8913, and 0.9439, respectively. Therefore, the Higuchi model was chosen and the time required for total release of the drug was calculated to be about 9 hours (the Higuchi model equation was *y* = 5.3491*x* − 5.8186, with *r* ^2^ = 0.9439). Therefore, the stability of the optimized nanospheres in simulated lung fluid (Gamble's solution) [16] regarding the particle size, zeta potential, and drug loading percent was studied after 9 hours which was a time period almost necessary for releasing of the total loaded drug from the nanospheres. The composition of the Gamble's solution included magnesium chloride 0.095 g/lit, sodium chloride 6.019 g/lit, potassium chloride 0.298 g/lit, disodium hydrogen phosphate (Na_2_HPO_4_) 0.126 g/lit, sodium sulfate 0.063 g/lit, calcium chloride dehydrate 0.368 g/lit, sodium acetate 0.574 g/lit, sodium hydrogen carbonate (NaHCO_3_) 2.604 g/lit, sodium citrate dihydrate 0.097 g/lit, and pH 7.4 [16].

## 3. Results and Discussion

### 3.1. Preparation of Agar Nanoparticles

The exceptional property of agar solution, that is, gel/sol transition at about 85°C and backward sol/gel transition at 35–40°C, makes the basis of its use in formation of micro- and nanoparticles. According to Maa and Hsu [31] and Tacholakova et al. [32], during emulsification by homogenization, equilibrium state is reached within 3 to 4 minutes of homogenization. With respect to preliminary experiments, two minutes of homogenization was decided to provide a satisfactory load while reducing the size down to nanofield.

Since these particles are formed out of a primary w/o emulsion, they are expected to have spherical shape. The presence of some agents, for example, cross-linkers in the formulation system, may somehow deviate the shape from a complete sphere.

As agar particles are very sticky, harvesting the particles and separating them in large amounts is a challenging task. They are not dissociated easily upon further reconstitution and this has compelled the scientist to obtain them in very low concentrations [17, 18]. To the best of our knowledge, nothing is mentioned in the literature on the final separation of the particles in large amounts so far. We have tried a simple technique consisting of adsorption of the final particles on mannitol powder. Washing the particles with ethanol helps to more rapid drying of the particles and inhibits their aggregation to a considerable extent.

### 3.2. Particle Size, Size Distribution, and Zeta Potential

The results of measuring of particle size, polydispersity index, and zeta potential of different formulations are seen in Table 1. The particle size of nanospheres obtained in different runs of the experiment was in the range of 310 nm (for C_10_S_12000_A_1_H_0_) to 807 nm (for C_6_S_8000_A_1.5_H_0.5_).

The obtained data were analyzed by the Design Expert Software according to the criteria mentioned in Section 2.7 and the optimum conditions for production of the nanospheres were suggested by the software.  Table 2 shows the results of regression analysis for particle size, zeta potential, drug release, and drug loading efficiency fitted to the two-factor model (in the two former cases), quadratic and linear equations, respectively. As seen in Table 2, the insignificant lack of fit (*P* > 0.05) shows the suitability of the fitted models. Positive sign (+) represents a synergistic effect on the response, while a negative sign (−) means an antagonist relationship. Phrases composed of two factors indicate the interaction terms and phrases with second-order factors stand for the nonlinear relationship between the response and the variable.

As Table 2 indicates, all studied models fitted to data were significant (*P* < 0.05) except for particle size of nanospheres (*P* > 0.05) and, among the predicted responses, all coefficients for loading efficiency were significant (*P* < 0.05).  Table 2 also shows that the homogenization speed and agar content had a decreasing effect on the particle size while their interactions had an increasing effect on this response. Nevertheless, all these effects were insignificant (*P* > 0.05). CaCl_2_ concentration and HP*β*CD increased the zeta potential with a nonsignificant effect (*P* > 0.05), although their interaction reduced it significantly (*P* < 0.05).

The multiple correlation coefficients (*R* ^2^) of the model for particle size, zeta potential, drug loading, and RE_300_% explained by the model are shown in Table 3. Results show that the predicted *R* ^2^ is in reasonable agreement with the adjusted *R* ^2^, indicating the adequacy of the model to predict the responses.

**Figure 2:**
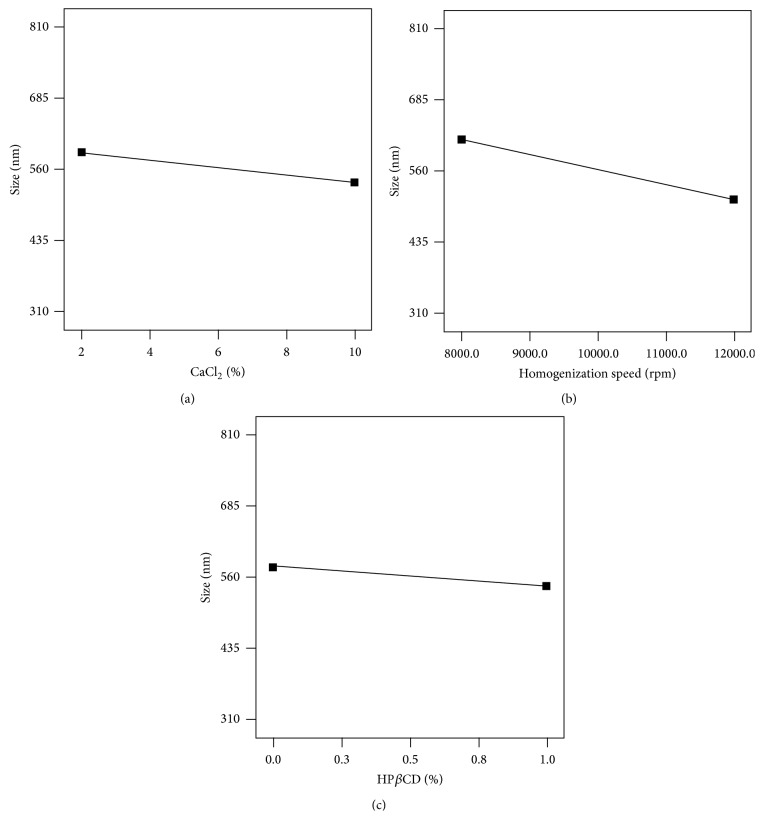
Correlation between particle size of agar nanospheres and (a) CaCl_2_%, (b) homogenizer speed, and (c) HP*β*CD%.

Figure 2 shows the effect of different studied variables on the particle of the nanospheres. Though the model relating the studied particle size to the input factors was not significant for any of the variables, there was a reduction in particle size with the increasing speed of homogenization (Figure 2) which was also reported in previous works [31]. According to Narsimhan and Goel [33, 34], the size of droplets in the dispersed phase during emulsification by homogenizer relates to counteraction of two opposite processes: breaking down of the droplets to smaller ones and coalescence of smaller droplets to form larger ones. The final size, that is, the ultimate equilibrium emulsion droplet size, depends upon the equilibration point between these two oppositely directed events.

The weak correlation (*P* = 0.0626) of particle size with speed of homogenization (Figure 2) in this study can be explained with the fact that the whole range of applied speed in this study, that is, 8000 rpm to 12000 rpm, was probably around the ultimate equilibrium emulsion droplet size.

Slight decrease in particle size or negative effect of CaCl_2_, as the cross-linking agent of agar, on the particle size of nanospheres (Table 2, Figure 2) can be due to partial center directed contraction that is exerted upon the spheres by the cross-linker.

Increasing the amounts of HP*β*CD led to some insignificant decrease in particle size (Figure 2) which can be due to the amphoteric structure of HP*β*CD, bestowing some surface acting property on it and finally reducing the droplet size of the primary emulsion.

As can be expected, change in agar contents of the emulsion had no effects on droplet size (Figure 2).

**Figure 3:**
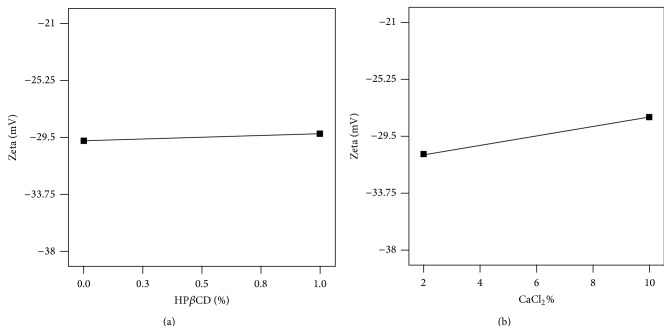
Correlation between zeta potential of agar nanospheres and (a) HP*β*CD% and (b) CaCl_2_%.

Cross-linking of the agar with calcium ions partially neutralizes the negative charge of the agar molecules and subsequently diminishes the absolute measure of zeta potential (Figure 3). Increasing amounts of HP*β*CD by covering more and more surface of the nanospheres through interaction with nonpolar projected groups can decrease the surface polar density which is finally translated into the decreasing of absolute amounts of zeta potential (Figure 3).

### 3.3. Morphology of Nanospheres

**Figure 4:**
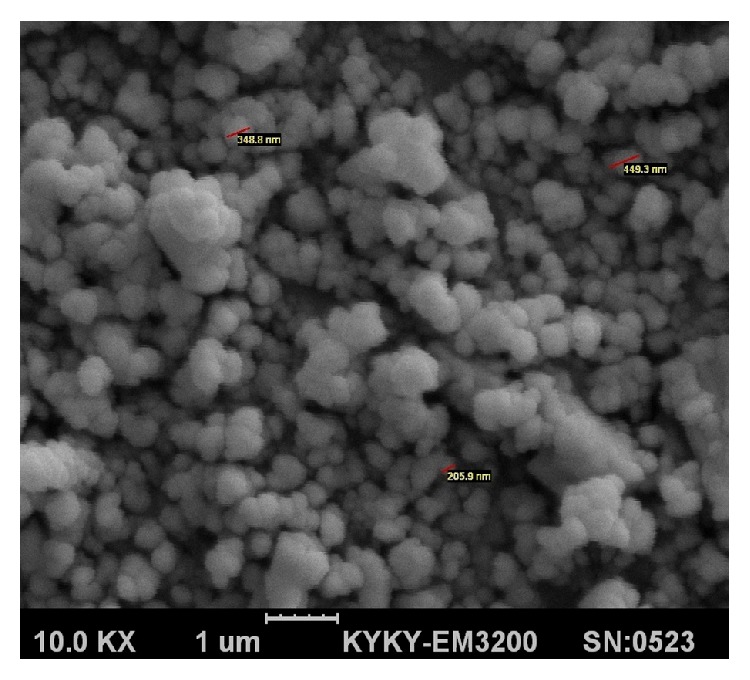
Scanning electron micrograph of agar nanospheres loaded with bupropion HCl.

As could be seen in Figure 4, the nanoparticles are almost spherical with acceptable uniformity. Trivial deviation from complete sphericity could be assigned to stresses exerted upon the nanospheres during congealing, for example, by the shear stress applied by the homogenizer or as a result of cross-linking. The SEM also confirms the particle size range already determined by zetasizer between 200 and 500 nm.

### 3.4. Drug Loading Efficiency

The results of measuring of drug loading efficiency of different formulations are seen in Table 1. As bupropion HCl is soluble in oily phase as well as in aqueous phase (log⁡*K*_o/w_ = 3.21 for bupropion), it is not unexpected to leave the agar solution during emulsification into the paraffin phase. Optimizing the time of homogenization has been tried to obtain acceptable load.

The results of Table 2 show that bupropion loading efficiency in the nanospheres was enhanced by increasing the agar content significantly (*P* < 0.05) while an antagonist effect was seen by the HP*β*CD (*P* < 0.05).

**Figure 5:**
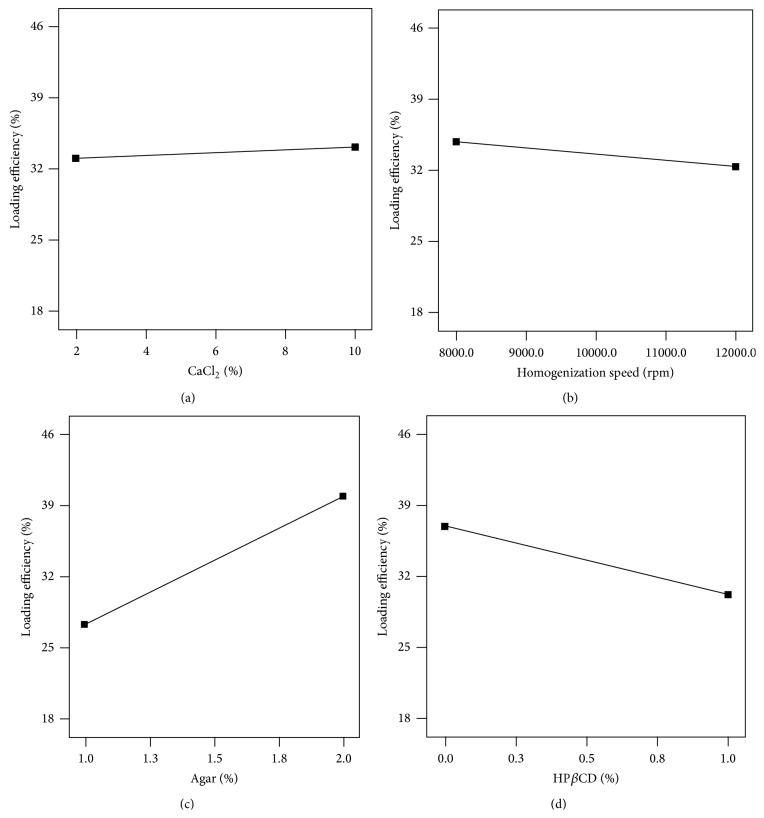
Effect of (a) CaCl_2_%, (b) homogenizer speed, (c) agar content, and (d) HP*β*CD% on drug loading efficiency in agar nanospheres.

As for the relationship between loading efficiency with input factors, calcium chloride caused more intimate entanglement of agar threads and constricted the pores through which bupropion molecules could not escape into the external phase during homogenizing. This in turn resulted in a higher loading efficiency (Figure 5). Higher speed of homogenizer by reducing the droplet size provided a larger surface for passing of the bupropion molecules into the oil phase and thus reduced the loading efficiency (Figure 5). HP*β*CD probably played the role of a vehicle to transport the active molecules into the paraffin which again decreased the drug loading (Figure 5). Agar presented an agonist effect on the drug loading efficiency in the nanospheres (Table 2) which obviously meant that more agar could accommodate more drug in the nanospheres and this was the strongest or the most significant correlation between the input variables and the output or the responses (Figure 5).

### 3.5. *In Vitro* Drug Release Studies

**Figure 6:**
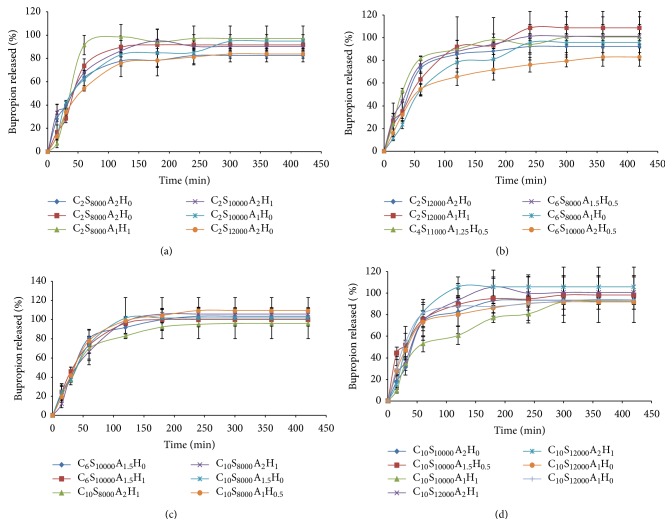
Release profiles of all 25 run, arranged based on the increasing amounts of CaCl_2_% and homogenizer speeds (the input factors with greater effects on release) from (a) to (d).

Release efficiency after 300 minutes was calculated for all 25 formulations (Table 1). The minimum RE was 63% (for C_6_S_10000_A_2_H_0.5_) and the maximum was 91% (for C_10_S_12000_A_2_H_1_). Bupropion release profiles of all 25 formulations are shown in (Figures 6(a) to 6(d)).

The results of Table 2 show that RE_300_% of the drug was increased insignificantly (*P* > 0.05) by the CaCl_2_ percent and homogenization speed while it was decreased by the agar content (*P* < 0.05).

**Figure 7:**
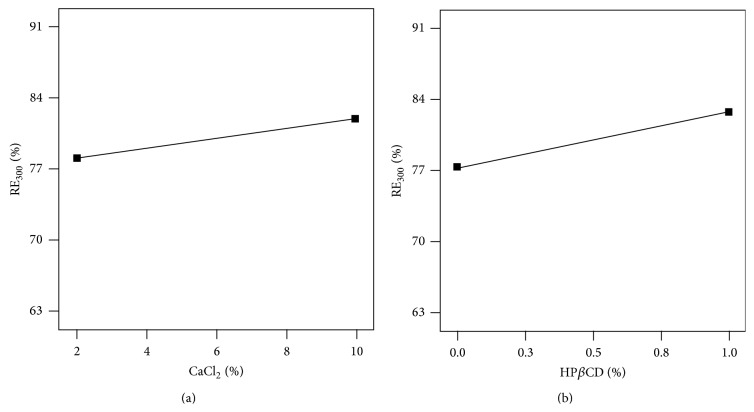
Variation of release efficiency of bupropion HCl from agar nanospheres with (a) CaCl_2_% and (b) HP*β*CD%.

RE_300_ showed a mild increase with increasing amount of cross-linking agent (Figure 7) that can be explained by axial squeezing out of bupropion towards the periphery of the nanospheres as the result of inward contraction exerted by the cross-linker which in turn means increasing accumulation of bupropion in the peripheral area of the matrix of the nanospheres and more rapid release.

As Figure 7 shows, more contents of HP*β*CD caused more facilitated entrance of bupropion into the release medium which may be due to the solubilizing nature of HP*β*CD causing greater RE_300_ (Figure 7).

### 3.6. Optimization

Numerical solution proposed by the software with the greatest desirability for the optimum formulation consisted of 7.19% calcium chloride, homogenization speed of 8500 rpm, 2% of agar, and 0.12% of HP*β*CD. Response factors corresponding to these inputs, predicted by the software, should show particle size of 577.68 nm, zeta potential of −30 mV, loading efficiency of 43.5%, and RE_300_ of 69.49%. Comparison between the predicted and real values of these responses and their error percent is summarized in Table 4.

### 3.7. Mucoadhesion Evaluation

The optimized nanospheres were tested for mucoadhesion which showed that the adsorption of mucin solution on to the nanospheres was nearly complete (99.5%). Therefore, it may be expected that these nanospheres will show strong mucoadhesion when applied to mucous and biomembranes of pulmonary route. Although the mucoadhesion test was carried out in acetate buffer due to the better dissolution of mucin, repeating of the test in simulated lung fluid (Gamble's Solution [16]) showed the mucoadhesion of 92.86% which was comparable to 99.5% obtained in acetate buffer. Some authors have reported that the airway surface liquid is slightly acidic, and this acidity might be part of normal airway defense. The pH value as low as 5.7 has been reported for the airway surface liquids by some authors [35]. Thus, pH of 4.5 in evaluating the mucoadhesion does not seem to adversely affect the results. Also, the presence of acetate ion in a majority of simulated lung fluids is mentioned in the literature [16].

As our preliminary studies showed, hydroxypropyl *β*-cyclodextrin did not show significant effect on the mucoadhesion of the nanospheres. However, to study its effect on the mucoadhesion of the optimized formulation, it was prepared with various concentrations of hydroxypropyl *β*-cyclodextrin (0, 0.5 and 1%) and the mucoadhesion of the optimized nanospheres was measured in acetate buffer. The results are shown in Table 5. As this table indicates, the different concentrations of HP*β*CD were not effective on the mucoadhesion of the nanospheres (*P* > 0.05).

### 3.8. Stability of the Nanospheres in the Simulated Lung Fluid

In the present work the nanospheres suspension is supposed to be administered into the rat lungs by using a micro-sprayer device which renders the suspension into micro-droplets capable to reach the deepest parts of pulmonary tract. Therefore, the stability of the nanoparticles was studied in the simulated lung fluid during the time they reside there and release their drug content. The results of stability studies of nanospheres in simulated lung fluid (Gamble's solution [16]) are shown in Table 6. The absence of drug degradation or great increase of particle size and zeta potential of nanospheres shows its good stability during its life span and residence time in the lungs. Although the measurements of these parameters at zero time in the water and simulated lung fluid are different (Table 6), the changes after 9 hours in the simulated lung fluid does not show significant difference (*P* > 0.05). The absence of many size increments during the life span of the nanospheres in the simulated lungs' fluid along with their suitable zeta potential of −11.6 mV which is high enough to prevent the aggregation of the nanospheres guarantees their stability in the lung during the period of their drug release.

## 4. Conclusions

Loading of bupropion HCl into agar nanospheres was performed successfully. Along with the active ingredient, cross-linking agent and a permeability increasing agent (HP*β*CD) were included in the nanospheres. Agar being a biodegradable/bioadhesive natural polymer is an attractive candidate for delivery of drugs in form of nanospheres via various biomembranes including pulmonary route. The relatively sufficient duration of release time (about 5 hours) as well as mucoadhesiveness of the nanospheres insures a considerable residence time of the nanospheres in vicinity of mucous membranes. The problem of stickiness of resulting agar nanospheres was overcome by freeze-drying or by simply adsorbing the nanospheres on mannitol powder. The optimum levels for studied independent factors proposed by the a D-optimal design were 7.19% of calcium chloride, homogenization speed of 8500 rpm, 2% of agar, and 0.12% of HP*β*CD. Response factors corresponding to these inputs, predicted by the software, were particle size of 577.68 nm, zeta potential of −30 mV, loading efficiency of 43.5%, and RE_300_ of 69.49%. Error percentages which give an estimate of the accordance between predicted and real values for the response factors were 1.61% and 2.65% for particle size and zeta potential, respectively, which are excellent. For loading efficiency, it was 11.26% which is rather acceptable but the error percent for RE_300_ is quiet large, that is, 26.6%. However, the D-optimal design turns out to be a somewhat proper method to optimize the fabrication of agar nanospheres though with some limitations markedly apparent for release efficiency. Agar nanospheres also showed excellent bioadhesiveness* in vitro*. To demonstrate the effectiveness of the designed nanospheres in the pulmonary delivery of bupropion, more detailed studies are performing on animal model* in vivo.*

## Figures and Tables

**Table 1:** Design of the experiment according to a D-optimal design for formulating agar nanospheres loaded with bupropion HCl.

Formulation	CaCl_2_ (%)	Homogenizer speed (rpm)	Agar (%)	HP*β*CD (%)	Particle size (nm)	Zeta potential (mV)	Loading efficiency (%)	RE_300_ (%)	PdI
C_10_S_8000_A_2_H_1_	10	8000	2	1	534.5 ± 18.6	−31.5	34.7 ± 0.3	90 ± 3	0.22
C_6_S_10000_A_2_H_0.5_	6	10000	2	0.5	716.2 ± 163.7	−32.8	31.4 ± 1.1	63 ± 6	0.62
C_10_S_8000_A_2_H_1_	10	8000	2	1	525.9 ± 63.7	−30.2	41.5 ± 1.2	79 ± 4	0.54
C_10_S_12000_A_1_H_0_	10	12000	1	0	613.4 ± 146.1	−27.2	29.4 ± 1.8	81 ± 10	0.56
C_2_S_8000_A_2_H_0_	2	8000	2	0	406.5 ± 79.6	−31.7	44.6 ± 2.7	70 ± 7	0.53
C_6_S_10000_A_1.5_H_0_	6	10000	1.5	0	503.9 ± 181.3	−31.0	35.4 ± 2.2	86 ± 8	0.45
C_2_S_10000_A_2_H_1_	2	10000	2	1	504.5 ± 114.8	−28.0	38.9 ± 0.9	80 ± 8	0.99
C_6_S_8000_A_1.5_H_0.5_	6	8000	1.5	0.5	807.2 ± 86	−25.9	39.5 ± 1.5	84 ± 5	0.51
C_2_S_12000_A_1_H_1_	2	12000	1	1	491.3 ± 29.9	−22.9	18.9 ± 2.0	84 ± 26	0.38
C_10_S_10000_A_1.5_H_0.5_	10	10000	1.5	0.5	492.3 ± 54.9	−25.4	34.9 ± 2.3	84 ± 17	0.9
C_10_S_12000_A_1_H_0_	10	12000	1	0	310.3 ± 10.4	−21.7	27.7 ± 1.0	77 ± 7	0.33
C_2_S_8000_A_1_H_1_	2	8000	1	1	659.1 ± 80.2	−29.9	20.8 ± 0.7	86 ± 9	0.21
C_6_S_8000_A_1_H_0_	6	8000	1	0	670.1 ± 159.5	−27.9	18.9 ± 0.4	72 ± 8	0.73
C_2_S_12000_A_2_H_0_	2	12000	2	0	519.7 ± 74.3	−34.2	43.6 ± 0.3	78 ± 4	0.48
C_2_S_8000_A_2_H_0_	2	8000	2	0	713.4 ± 97.3	−35.2	40.0 ± 1.0	78 ± 3	0.42
C_10_S_12000_A_2_H_1_	10	12000	2	1	494.5 ± 19.3	−29.8	36.3 ± 0.3	91 ± 6	0.52
C_10_S_8000_A_1_H_0.5_	10	8000	1	0.5	723.2 ± 304.7	−24.6	32.1 ± 0.5	87 ± 2	0.61
C_6_S_10000_A_1.5_H_1_	6	10000	1.5	1	455.3 ± 153.3	−31.3	37.2 ± 0.6	86 ± 3	0.69
C_10_S_10000_A_2_H_0_	10	10000	2	0	453.2 ± 19	−30.0	44.3 ± 0.9	78 ± 2	0.25
C_10_S_8000_A_1.5_H_0_	10	8000	1.5	0	619.9 ± 132.2	−28.0	45.6 ± 2.2	87 ± 10	0.63
C_4_S_11000_A_1.25_H_0.5_	4	11000	1.25	0.5	443.4 ± 22.9	−34.7	31.7 ± 0.7	85 ± 4	0.72
C_10_S_12000_A_2_H_1_	10	12000	2	1	674.1 ± 79.1	−31.7	30.0 ± 1.1	87 ± 5	0.47
C_10_S_10000_A_1_H_1_	10	10000	1	1	357.3 ± 7.9	−29.0	26.6 ± 1.3	65 ± 4	0.58
C_2_S_12000_A_2_H_0_	2	12000	2	0	641.7 ± 32.0	−27.3	44.1 ± 2.4	67 ± 5	0.51
C_2_S_10000_A_1_H_0_	2	10000	1	0	776.6 ± 50.1	−37.1	36.6 ± 1.1	74 ± 4	0.54

^*^C: CaCl_2_, S: speed of homogenizer, A: agar, and H: hydroxyl *β*-cyclodextrin.

**Table 2:** Regression analysis for particle size, zeta potential, drug loading efficiency, and RE_300_%.

Responses												
Factor	Particle size			Zeta potential			Drug loading efficiency %			RE_300_%		
	C.E	Std. error	*P* value	C.E	Std. error	*P* value	C.E	Std. error	*P* value	C.E	Std. error	*P* value
Int	+561.70	24.58	0.1004	−29.37	0.64	0.0118	+33.70	1.03	<0.0001	+81.31	3.06	0.0289
*X*_1_	—	—	—	+1.27	0.73	0.0969	—	—	—	+0.84	1.48	0.5760
*X*_2_	−60.02	30.53	0.0626	—	—	—	—	—	—	+0.73	1.59	0.6516
*X*_3_	−3.45	27.94	0.9030	—	—	—	+6.31	1.18	<0.0001	−0.28	1.43	0.8456
*X*_4_	—	—	—	+0.34	0.72	0.6386	−3.21	1.15	0.0106	—	—	—
*X*_1_^2^	—	—	—	—	—	—	—	—	—	5.29	3.70	0.1708
*X*_2_^2^	—	—	—	—	—	—	—	—	—	5.88	2.88	0.0565
*X*_3_^2^	—	—	—	—	—	—	—	—	—	−11.87	3.70	0.0052
*X*_1_ *X*_3_	—	—	—	—	—	—	—	—	—	3.87	1.52	0.0211
*X*_1_ *X*_4_	—	—	—	−2.41	0.78	0.0053	—	—	—	—	—	—
*X*_2_ *X*_3_	+66.11	32.73	0.0563	—	—	—	—	—	—	—	—	—

Lack of fit values												
*F* value	0.52			1.05			2.78			1.18		
*P* value	0.8524			0.5270			0.1315			0.4595		

*X*_1_: CaCl_2_ percent, *X*_2_: homogenization speed, *X*_3_: agar percent, *X*_4_: HP*β*CD percent, C.E: coefficient estimate in terms of actual factors, and Int: intercept; the positive sign of the factor represents a synergistic effect on the response, while a negative sign means an antagonist relationship.

**Table 3:** Summary of results of regression analysis for responses *Y*_1_, *Y*_2_, *Y*_3_, and *Y*_4_.

Model	*R* ^2^	Adjusted *R* ^2^	Predicted *R* ^2^	Mean	S.D.	C.V.%
*Y*_1_ (particle size)	0.2522	0.1454	−0.0439	564.30	121.77	21.58
*Y*_2_ (Zeta potential)	0.4004	0.3147	0.1353	−29.56	3.14	10.63
*Y*_3_ (drug loading efficiency)	0.6135	0.5783	0.5034	34.59	5.11	14.78
*Y*_4_ (RE_300_%)	0.5559	0.3731	0.0260	79.96	6.16	7.71

**Table 4:** Predicted versus real response factors for the optimum formulation (measured in deionized and purified water).

	Particle size (nm)	Zeta potential (mV)	Loading efficiency (%)	RE_300_ (%)
Predicted	577.68	−30.10	43.5	69.49
Real	587 ± 58	−30.9	38.6 ± 1.3	51 ± 9
Error percent	1.61	2.65	11.26	26.6

**Table 5:** The results of mucoadhesion measurement of the optimized agar nanospheres in different fluids and in different concentrations of hydroxypropyl *β*-cyclodextrin (in acetate buffer).

Acetate buffer	Simulated lung fluid	HP*β*CD 0%	HP*β*CD 0.5%	HP*β*CD 1%
99.5%	92.86%	99.4%	99.5%	99.3%

**Table 6:** The results of measuring the particle size, zeta potential, and drug content of the optimum formulation measured in the simulated lung fluid [16] immediately and 9 hours after preparation.

Particle size (nm)		Zeta potential (mV)	
At zero time	After 9 hrs	At zero time	After 9 hrs
553 ± 32	600 ± 40	−11.6	−10.6

## References

[1] Casey Laizure S., Lindsay DeVane C., Stewart J. T., Dommisse C. S., Lai A. A. (1985). Pharmacokinetics of bupropion and its major basic metabolites in normal subjects after a single dose. *Clinical Pharmacology and Therapeutics*.

[2] Brunton L. L., Lazo J. S., Parker K. L. (2006). *Goodman & Gilman’s the Pharmacological Basis of Therapeutics*.

[3] Stahl S. M., Pradko J. F., Haight B. R., Modell J. G., Rockett C. B., Coughlin S. L. (2004). A review of the neuropharmacology of bupropion, a dual norepinephrine and dopamine reuptake inhibitor. *The Primary Care Companion—Journal of Clinical Psychiatry*.

[4] Mehta N. B. (1974). Meta-chloro-substituted alpha-butylaminopropiophenones. *US Patent*.

[5] Sweet R. A., Pollock B. G., Kirshner M., Wright B., Altieri L. P., DeVane C. L. (1995). Pharmacokinetics of single- and multiple-dose bupropion in elderly patients with depression. *Journal of Clinical Pharmacology*.

[6] Suckow R. F., Smith T. M., Perumal A. S., Cooper T. B. (1986). Pharmacokinetics of bupropion and metabolites in plasma and brain of rats, mice, and guinea pigs. *Drug Metabolism and Disposition*.

[7] Zung W. W. K., Brodie H. K. H., Fabre L., McLendon D., Garver D. (1983). Comparative efficacy and safety of bupropion and placebo in the treatment of depression. *Psychopharmacology*.

[8] Jorenby D. E., Hays J. T., Rigotti N. A. (2006). Efficacy of varenicline, an *α*4*β*2 nicotinic acetylcholine receptor partial agonist, vs placebo or sustained-release bupropion for smoking cessation: a randomized controlled trial. *Journal of the American Medical Association*.

[9] Labbate L. A., Grimes J. B., Hines A., Pollack M. H. (1997). Bupropion treatment of serotonin reuptake antidepressant-associated sexual dysfunction. *Annals of Clinical Psychiatry*.

[10] Plodkowski R. A., Nguyen Q., Sundaram U., Nguyen L., Chau D. L., St Jeor S. (2009). Bupropion and naltrexone: a review of their use individually and in combination for the treatment of obesity. *Expert Opinion on Pharmacotherapy*.

[11] Cantwell D. P. (1997). ADHD through the life span: the role of bupropion in treatment. *The Journal of Clinical Psychiatry*.

[12] Modell J. G., Rosenthal N. E., Harriett A. E. (2005). Seasonal affective disorder and its prevention by anticipatory treatment with bupropion XL. *Biological Psychiatry*.

[13] Brody A. L., Mukhin A. G., Mamoun M. S. (2013). Treatment for tobacco dependence: effect on brain nicotinic acetylcholine receptor density. *Neuropsychopharmacology*.

[14] Zhang D., Yuan B., Qiao M., Li F. (2003). HPLC determination and pharmacokinetics of sustained-release bupropion tablets in dogs. *Journal of Pharmaceutical and Biomedical Analysis*.

[15] Silverstone P. H., Williams R., McMahon L., Fleming R., Fogarty S. (2008). Convulsive liability of bupropion hydrochloride metabolites in Swiss albino mice. *Annals of General Psychiatry*.

[16] Marques M. R., Loebenberg R., Almukainzi M. (2011). Simulated biological fluids with possible application in dissolution testing. *Dissolution Technologies*.

[17] Wang N., Wu X. S. (1997). Preparation and characterization of agarose hydrogel nanoparticles for protein and peptide drug delivery. *Pharmaceutical Development and Technology*.

[18] Ju L. E., Park J. K., Khan S. A., Lim K. H. (2011). Preparation of agar nanoparticles by W/O emulsification. *Journal of Chemical Engineering of Japan*.

[19] Wang N., Wu X. S. (1998). A novel approach to stabilization of protein drugs in poly(lactic-co-glycolic acid) microspheres using agarose hydrogel. *International Journal of Pharmaceutics*.

[20] Bera K., Sarwa K. K., Mazumder B. (2013). Metformin HCl loaded mucoadhesive agar microspheres for sustained release. *Asian Journal of Pharmaceutics*.

[21] Bilati U., Allémann E., Doelker E. (2005). Development of a nanoprecipitation method intended for the entrapment of hydrophilic drugs into nanoparticles. *European Journal of Pharmaceutical Sciences*.

[22] Selby H. H., Wynne W. H., Whistler R. L. (1973). Agar. *Industrial Gums*.

[23] Manjunatha K., Ramana M., Satyanarayana D. (2007). Design and evaluation of diclofenac sodium controlled drug delivery systems. *Indian Journal of Pharmaceutical Sciences*.

[24] Hjertén S. (1964). The preparation of agarose spheres for chromatography of molecules and particles. *Biochimica et Biophysica Acta—Specialized Section on Biophysical Subjects*.

[25] Mu Y., Lyddiatt A., Pacek A. W. (2005). Manufacture by water/oil emulsification of porous agarose beads: effect of processing conditions on mean particle size, size distribution and mechanical properties. *Chemical Engineering and Processing: Process Intensification*.

[26] Egorov A. M., Vakhabov A. K., Chernyak V. Y. (1970). Isolation of agarose and granulation of agar and agarose gel. *Journal of Chromatography A*.

[27] Linghui Z., Ling-ling Y., Ling-min Z., Xing-long J., Xun-Qing T., Ji-Ye C. http://www.paper.edu.cn/.

[28] Tewes F., Gobbo O. L., Amaro M. I. (2011). Evaluation of HP*β*CD-PEG microparticles for salmon calcitonin administration via pulmonary delivery. *Molecular Pharmaceutics*.

[29] Loboz K. K., Gross A. S., Ray J., McLachlan A. J. (2005). HPLC assay for bupropion and its major metabolites in human plasma. *Journal of Chromatography B: Analytical Technologies in the Biomedical and Life Sciences*.

[30] Varshosaz J., Dehghan Z. (2002). Development and characterization of buccoadhesive nifedipine tablets. *European Journal of Pharmaceutics and Biopharmaceutics*.

[31] Maa Y.-F., Hsu C. (1996). Liquid-liquid emulsification by rotor/stator homogenization. *Journal of Controlled Release*.

[32] Tcholakova S., Denkov N. D., Sidzhakova D., Ivanov I. B., Campbell B. (2003). Interrelation between drop size and protein adsorption at various emulsification conditions. *Langmuir*.

[33] Narsimhan G., Goel P. (2001). Drop coalescence during emulsion formation in a high-pressure homogenizer for tetradecane-in-water emulsion stabilized by sodium dodecyl sulfate. *Journal of Colloid and Interface Science*.

[34] Narsimhan G. (2004). Model for drop coalescence in a locally isotropic turbulent flow field. *Journal of Colloid and Interface Science*.

[35] Fischer H., Widdicombe J. H. (2006). Mechanisms of acid and base secretion by the airway epithelium. *Journal of Membrane Biology*.

